# Pyrogen detection methods: Comparison of bovine whole blood assay (bWBA) and monocyte activation test (MAT)

**DOI:** 10.1186/2050-6511-15-50

**Published:** 2014-09-10

**Authors:** Christian Wunderlich, Stephan Schumacher, Manfred Kietzmann

**Affiliations:** 1 University of Veterinary Medicine Hannover, Foundation, Institute of Pharmacology, Toxicology and Pharmacy, Bünteweg 17, Hannover 30559, Germany

**Keywords:** Endotoxin, Bovine whole blood, Prostaglandin E_2_, Pyrogen, Monocyte activation test, Lipopolysaccharide, Lipoteichoic acid

## Abstract

**Background:**

Pyrogen detection is of utmost importance in pharmaceutical industry, laboratories and health care institutions. As an alternative to the animal-consuming rabbit pyrogen test or Limulus amoebocyte lysate test, the monocyte activation test was introduced as a gold standard method in the European Pharmacopoeia. However, the monocyte activation test has not gained wide acceptance in practice.

**Methods:**

We stimulated bovine whole blood with different endotoxin preparations (lipopolysaccharide *E.coli* 0127:B8 and 0113:H10), as well as the non-endotoxin pyrogens peptidoglycan and lipoteichoic acid. Prostaglandin E_2_ (PGE_2_) served as read out.

**Results:**

Employing PGE_2_ as read out enabled detection limits of 0.04 EU/ml for lipopolysaccharide 0127:B8, 0.25 EU/ml for lipopolysaccharide 0113:H10 and 10 μg/ml of lipoteichoic acid as well as peptidoglycan. To evaluate the bWBA test system as a possible alternative to the MAT we performed a peer-to-peer comparison of the two methods and confirmed similar sensitivities.

**Conclusions:**

In conclusion, the bovine whole blood assay (bWBA) reproducibly enabled sensitive detection of endotoxin and non-endotoxin pyrogens and may thus become a viable alternative for pyrogen testing.

## Background

The detection of pyrogenic contamination is an essential part of drug safety testing in the pharmaceutical industry, reference laboratories as well as health care institutions. To guarantee patient safety, critical threshold levels of pyrogenic contamination have been determined and must not be exceeded. Therefore the European Pharmacopoeia (EP) promotes the monocyte activation test (MAT) as most suitable test for pyrogen testing [1]. Former methods, the rabbit pyrogen test (RPT) [2] and the Limulus amoebocyte lysate (LAL) test [3] are limited by inherent disadvantages since the RPT has a comparably low sensitivity for pyrogens [4] and the LAL is unable to detect non-endotoxin pyrogens [5]. Moreover, both are animal-consuming tests which, according to the 3 R concept – replacement, reduction, refinement –, should be avoided [6-8].

Nevertheless, product safety has to remain the first priority of medical product legislation, while economic considerations are also important to the industry and suitable methods need to offer a reasonable cost-benefit ratio. The MAT utilizes human blood [9,10] and is characterized by a high sensitivity for detecting endotoxin and non-endotoxin pyrogens. However, apparently it did not satisfy the needs of the pharmaceutical industry because it has not been widely used since its introduction in 2010. This might be partly due to the fact that accessing fresh human whole blood or producing large amounts of cryoblood of uniform quality for use in the MAT is certainly a logistic challenge. According to the European Pharmacopoeia, blood donors must confirm that they have been free of signs of infection and have not taken anti-inflammatory medications for one week before donation [1]. Additionally, commercialized cryoblood is routinely tested for sterility and HIV, HAV, HCV and HBV [11]. However, there remain several potentially influencing factors that cannot be standardized in a human-based test system since lifestyle and genetic background certainly differ significantly between donors.

In principal, if blood from a large animal species and a designated breed would be used for pyrogen detection most of these limitations could be overcome, because the animals can be housed under standardized specific pathogen-free conditions. Several aspects favor the use of bovine blood, for instance the fact that the Toll-like receptor equipment of bovine leukocytes is comparable to humans [12] as well as reports suggesting the suitability of bovine blood for the detection of lipopolysaccharides (LPS) [13].

In a previous study we reported that bovine whole blood can be used for a sensitive detection of LPS 0111:B4 from *E.coli* by using Prostaglandin E_2_ as readout [14]. In the present study we investigated whether the system we established was also capable of detecting other endotoxins and gram-positive cell wall components. Additionally, we compared our method with the commercially available PyroDetect System (MAT) in a peer-to-peer setup.

## Methods

### Used stimulants

Endotoxin derived from *Escherichia coli* 0127:B8 (L3129, Sigma-Aldrich, Steinheim, Germany; stock ≥ 500000 EU/mg), WHO standard endotoxin from *Escherichia coli* 0113:H10:K (10 000 IU per vial, Merck, Darmstadt, Germany), peptidoglycan from *Bacillus subtilis* (low endotoxin, ≤ 1 EU/mg, InvivoGen, Toulouse, France) and lipoteichoic acid from *Staphylococcus aureus* (low endotoxin, ≤ 1 EU/mg, InvivoGen) were used as stimulants. Solutions were prepared with LAL-water or pyrogen-free saline. Aliqouts were stored at −20°C, except for the WHO Endotoxin, which was stored at −80°C. Immediately prior to use the aliquots were thawed, sonicated and diluted with pyrogen-free saline into different concentrations. Concentrations used for LPS 0127:B8 were 0.039, 0.078, 0.156, 0.313, 0.625 and 1.25 EU/ml, for LPS 0113:H10 0.063, 0.125, 0.25, 0.5, 1 and 2 EU/ml. Peptidoglycan and lipoteichoic acid were diluted to 1, 10, 50, 100 and 1000 μg/ml.

### Blood collection and ethical statement

Blood was obtained via venipuncture from healthy cattle (mainly Holsteins except two crossbreds and one red Holstein) into 7.5 ml heparinized tubes (Li-Heparin, 19 IU/ml, SARSTEDT Monovette, Nümbrecht, Germany). The animals were owned by and stabled in the Clinic for Cattle of the University of Veterinary Medicine Hannover, Foundation. All blood donors were female, non-lactating cows and were fed with hay ad libitum. The age ranged from 2.5 to 13 years.

This study received ethical approval by the Lower Saxony State Office for Consumer Protection and Food Safety (LAVES), Oldenburg (Az. 33.9-42502-05-13A361). All procedures involving animals were carried out in accordance with German legislation on animal welfare.

### *In vitro* assay using bovine peripheral blood

225 μl lithium heparin blood from different donors were pipetted into 96-well cell culture plates (SARSTEDT, Nümbrecht, Germany) and stimulated for 24 hours with 25 μl pyrogen solution or vehicle. After incubation at 37°C and 5% CO_2_, the 96-well plates were centrifuged at 2272 × g for 10 minutes, the supernatants were collected and frozen at −80°C until analysis. PGE_2_ concentration was determined using the Cayman Prostaglandin E_2_ Express EIA Kit (Cayman Chemical Company, Ann Arbor, MI, USA) following the manufacturer’s instructions.

### Comparison with the PyroDetect system

The PyroDetect System (Merck, Darmstadt, Germany) was used according to the manufacturer’s instructions as a quantitative test with the exception that samples were not tested at different dilutions. The quantitative test is described by the producers as method A. By using method A, a quantitative comparison of the samples with the standard endotoxin is possible. In summary, the stimulating agents were pipetted as 20 μl portions into the 96-well plate (included in the kit) under a horizontal flow bench. Apart from the spike and blank wells – the former was filled with stimulant and spiked RPMI medium, the latter with a total of 40 μl RPMI – 20 μl RPMI were added to each well. The two cryo blood vials (included in the kit) were thawed in a water bath for 1 minute and diluted immediately with 8 ml RPMI 1640 cell culture medium each (included in the kit). Afterwards the cryopreserved blood mixture was pipetted into the plate at a volume of 200 μl per well. After incubation for 16 hours at 37°C with 5% CO_2_ the mixture in the wells was resuspended five times and transferred to the ELISA plate (included in the kit). The IL-1β ELISA was performed following the manufacturer’s instructions. To compare this test system with the bovine whole blood assay the same stimulants (diluted in RPMI) were tested simultaneously using the blood of 6 animals (separately) following the method described before.

### Statistical analysis

Statistical analysis was carried out using the software SAS 9.3 (SAS, Cary, NC, USA). Data were checked for normal distribution by visual inspection and the Kolmogorov-Smirnov test. Some data sets showed a left-skewed distribution and failed the normality test. Therefore a permutation test (10000 permutations) was used for calculating a randomized complete block design (equivalent to exact Friedmann Test) and P values smaller 0.05 were considered significant. Calculations were done with the SAS macro RIBDPERM.MAC (provided by Erich Schumacher, Institut für Angewandte Mathematik und Statistik, Universität Hohenheim). Data are represented as box-plot with median and min to max whiskers.

## Results

### Pyrogen stimulation

**Figure 1:**
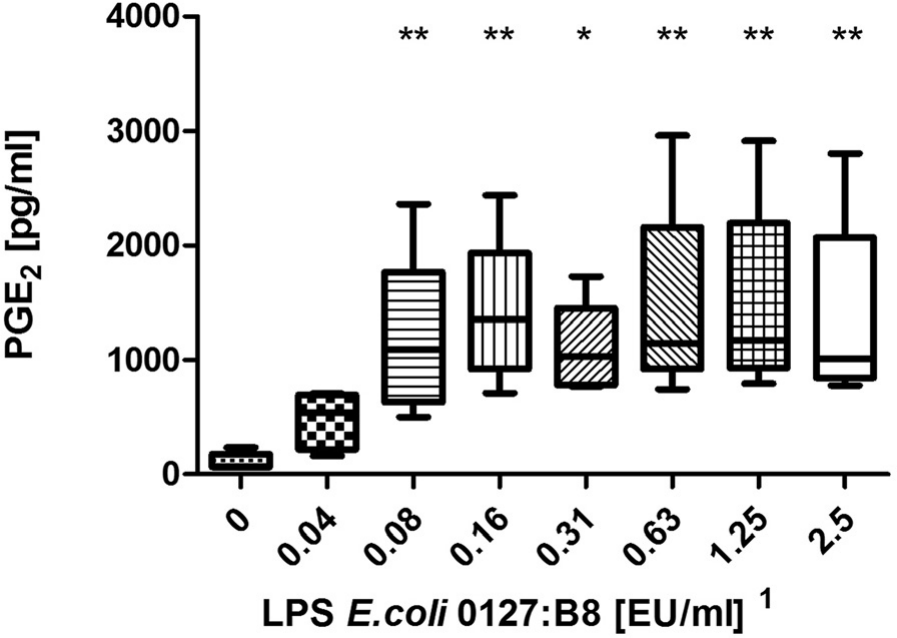
**Stimulation with LPS from E.coli 0127:B8.** Prostaglandin E_2_ concentration after LPS stimulation of fresh (stored < 2 h) bovine whole blood. Box-plot with median and min to max whiskers of n = 9, permutation test, *p ≤ 0.05, **p ≤ 0.01 compared to unstimulated blood. Result is representative of three independent experiments^1^.

**Figure 2:**
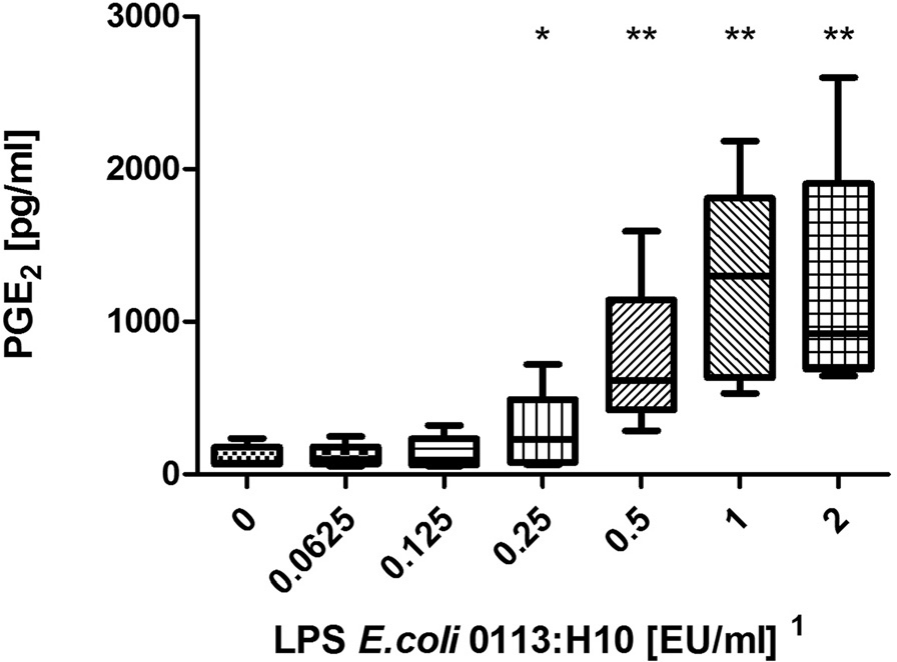
**Stimulation with WHO standard endotoxin.** Prostaglandin E_2_ concentration after LPS stimulation of fresh (stored < 2 h) bovine whole blood. Box-plot with median and min to max whiskers of n = 5, permutation test, *p ≤ 0.05, **p ≤ 0.01 compared to unstimulated blood. Result is representative of three independent experiments^1^.

**Figure 3:**
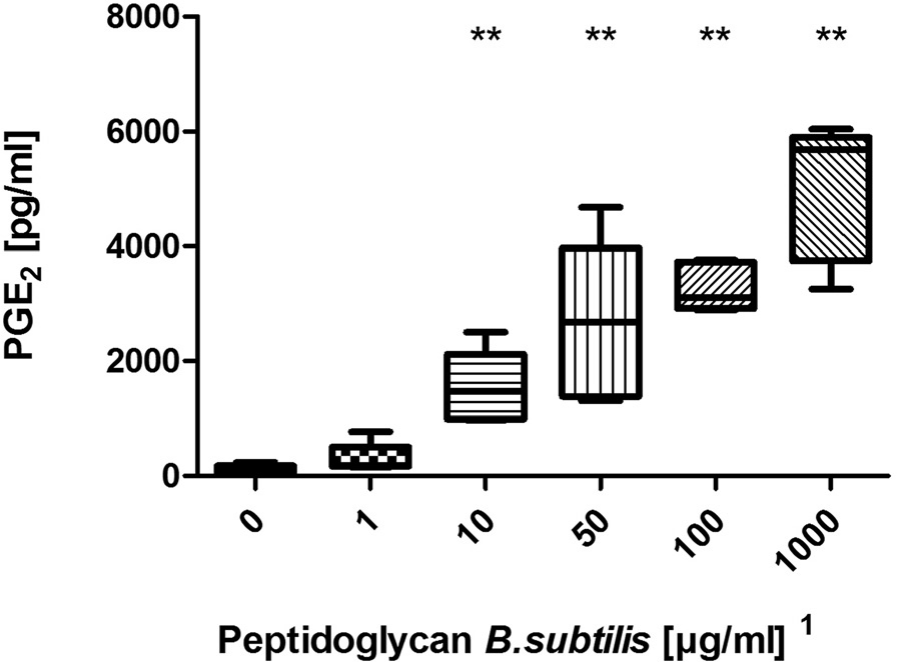
**Stimulation with peptidoglycan from Bacillus subtilis.** Prostaglandin E_2_ concentration after PGN stimulation of fresh (stored < 2 h) bovine whole blood. Box-plot with median and min to max whiskers of n = 5, permutation test, **p ≤ 0.01 compared to unstimulated blood. Result is representative of three independent experiments^1^.

**Figure 4:**
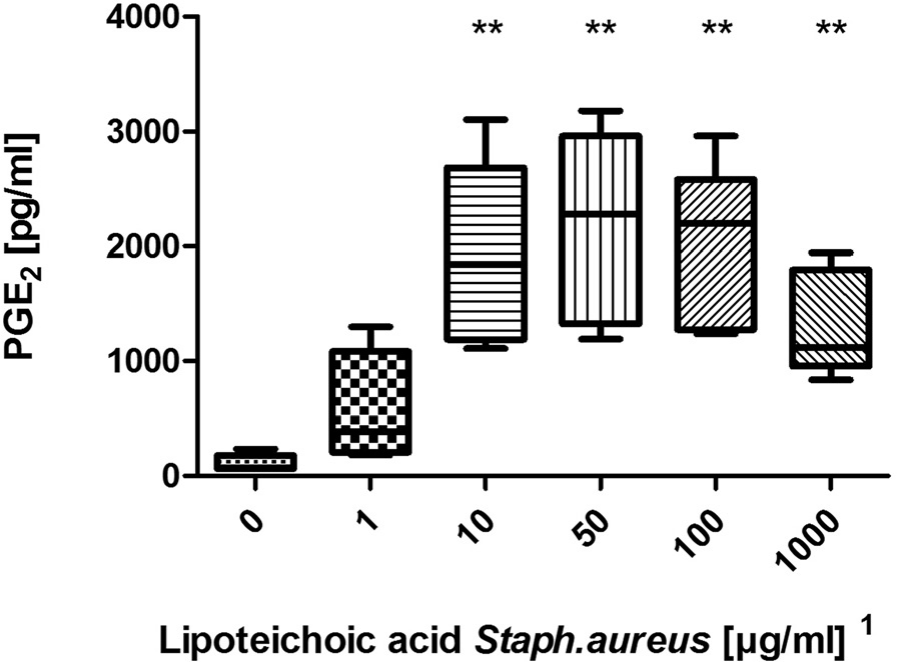
**Stimulation with lipoteichoic acid from Staphylococcus aureus.** Prostaglandin E_2_ concentration after LTA stimulation of fresh (stored < 2 h) bovine whole blood. Box-plot with median and min to max whiskers of n = 5, permutation test, **p ≤ 0.01 compared to unstimulated blood. Result is representative of three independent experiments^1^.

LPS from E.coli 0127:B8 was used as a stimulating agent and we found a dose-dependent increase of PGE_2_ starting at a dose of 0.08 EU/ml and reaching a plateau at 0.16 EU/ml (Figure 1). Using the WHO standard endotoxin (LPS E.coli 0113:H10) we discovered a dose-dependent increase of PGE_2_ release starting at 0.25 EU/ml (Figure 2). Peptidoglycan (PGN) from *Bacillus subtilis* induced a dose-dependent increase of PGE_2_ in concentrations of more than 10 μg/ml (Figure 3). Likewise, lipoteichoic acid (LTA) from *Staphylococcus aureus* provoked a significant increase of PGE_2_ at concentrations of 10 μg/ml and above (1 μg/ml provoked an increase as well, but fell just short of the level of significance, p = 0.056). The maximum PGE_2_ production was seen at 50 μg/ml, but although eicosanoid release elicited by higher LTA concentrations declined, it remained significantly higher compared to unstimulated blood (Figure 4).

### Bovine whole blood assay compared to PyroDetect system

**Figure 5:**
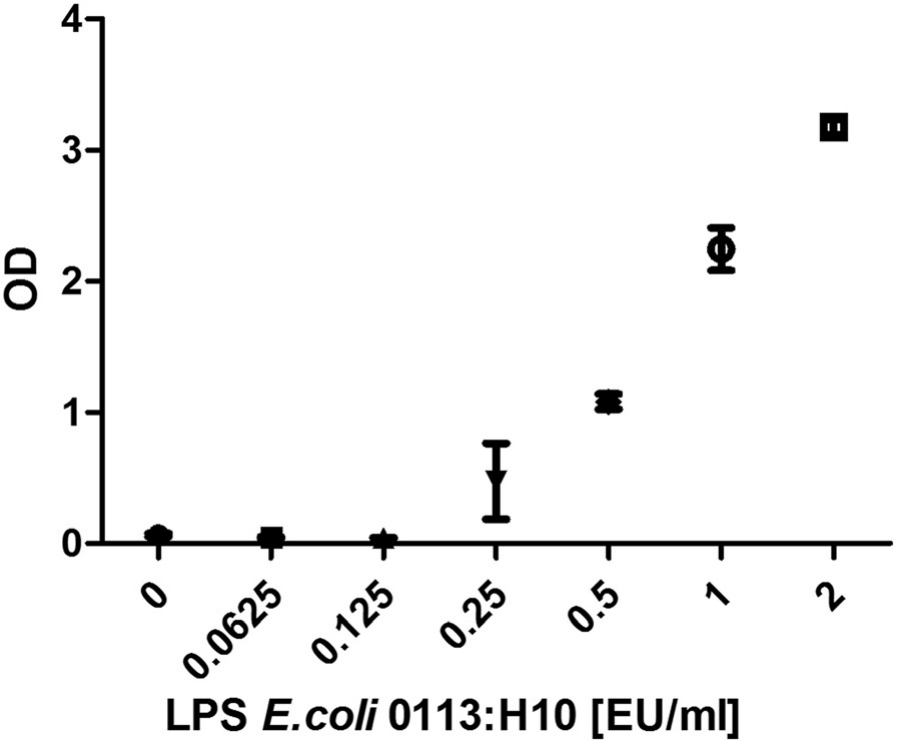
**PyroDetect System standard curve.** PyroDetect System WHO standard endotoxin calibration curve, optical density in relation to LPS concentration. Mean with SEM of pooled cryoconserved human blood in 4 measurements.

After 16 hours of stimulation – analogously to the manufacturer’s lab procedure – the PyroDetect System ELISA detected the presence of 0.25 EU/ml standard endotoxin (Figure 5), whereas 0.0625 EU/ml and 0.125 EU/ml did not induce measurable cytokine production. Importantly, the PyroDetect System also detected the presence of all other pyrogens at all concentrations used. Unfortunately, the color reaction of the ELISA was so intense that we were unable to quantify it thus precluding the specification of EU equivalents.

The simultaneous stimulation of bovine blood from 6 animals with the same pyrogens resulted in detection limits comparable to those obtained from the previous experiments. Results of one animal were excluded from analysis because of preexisting PGE_2_ release from the unstimulated blood. The comparative detection limits of the two test methods are given in Table 1.

## Discussion

We have previously shown that LPS 0111:B4 from *E.coli* can be reliably detected using a bovine whole blood assay and PGE_2_ as readout [14]. Hartung and Wendel [15] reported the suitability of a test method utilizing human whole blood for the detection of endotoxin as well as non-endotoxin pyrogens. The quite similar toll-like receptor (TLR) equipment of human and bovine leukocytes [12] gave reason to expect the same would be possible using our test system. Thus, we decided to investigate whether the bovine whole blood assay was capable of detecting a broad range of pyrogens with the required sensitivity.

### Detection of LPS

The bWBA was capable of detecting the presence of two different kinds of endotoxins. Remarkably, the detection of LPS 0127:B8 was 3 – 6 fold more sensitive than the detection of LPS 0113:H10 (0.04 – 0.08 EU/ml vs. 0.25 – 0.5 EU/ml) which was confirmed using the PyroDetect System. Similar findings were reported by others who showed an up to 1000-fold difference in potency of different endotoxins [16,17]. However, the observed detection limits of both test systems for the WHO standard endotoxin (LPS *E.coli* 0113:H10) still complied with the specifications of the MAT and the required sensitivity of pyrogen testing of 0.5 EU/ml [1].

### Detection of PGN

The bWBA was capable of detecting 10 – 50 μg/ml PGN. It is still a matter of controversy how the stimulatory potency of PGN is conveyed. Although it has long been thought to be an agonist of the TLR2, PGN is probably sensed by the nucleotide-binding oligomerization domain-containing proteins (NOD) 1 and 2 [18]. Moreover, it has been reported that commercial PGN preparations are often contaminated with endotoxin [19] and highly purified PGN was unable to stimulate cytokine release in a human whole blood test system [20,21]. This issue was also discussed in a meta-analysis of Rockel and Hartung [22] and to date there is no evidence that highly purified PGN is an immune stimulant. In order to prevent the effects of a potential endotoxin contamination we used a PGN preparation from Invivogen. This manufacturer guarantees an endotoxin content of less than 1 EU per milligram. Regarding that in our study detectable LPS concentrations were in the pg/ml range, whereas non-endotoxin pyrogens could be detected in the μg/ml range a falsification of the results due to LPS contamination should be unlikely although it cannot be completely excluded.

### Detection of LTA

The bWBA was capable of detecting 10 μg/ml LTA. Interestingly, we observed a decrease of PGE_2_ release at the highest concentrations of LTA, a finding which has not been reported in the literature so far. A possible explanation could be a complex formation of LTA at high concentrations [23], a phenomenon that seems to occur because of the intermolecular interactions of LTA molecules. Another explanation could be an enhanced binding by the bovine scavenger receptor type 1 [24], which has the ability to interact with or bind, for example, pyrogens. However, it remains unclear why this did not seem to apply to PGN. Yet, despite the decline of PGE_2_, the high concentrations of LTA still remained detectable and concentrations above 100 μg/ml are very unlikely to occur in pyrogen-contaminated medicinal products.

A meta-analysis by Rockel and Hartung discussed LTA as a possible reference stimulant for grampositive bacteria, analogous to endotoxin for gramnegative bacteria [22]. Similar to PGN some commercial LTA preparation have been reported to be contaminated by LPS [25], so again we used a preparation from Invivogen to reduce the risk of false positive results. Nevertheless, it is not clear whether LTA itself is a pyrogen or not. Zähringer et al. [18] elegantly elucidated how contaminating lipopetides like MALP-2 likely explain the TLR2-stimulating effects of LTA and PGN preparations. A TLR2-agonistic activity of natural and synthetic lipopeptides has also been confirmed by others [26].

### Comparison of the MAT and bWBA

In order to compare the sensitivity of the bovine whole blood assay with the commercially available PyroDetect System we decided to perform a peer-to-peer comparison with the same stimulants. The comparison of the two methods resulted in the same detection level of WHO standard endotoxin, 0.25 EU/ml – sufficing the postulated allowed level of 0.5 EU/ml in pharmaceutical products [1]. With regard to PGN, LTA and LPS 0127:B8 the PyroDetect System appeared to be more sensitive. It was capable of identifying the presence of 1 μg/ml PGN or LTA (the lowest concentrations used in our experiments) and other studies suggest that the MAT can detect concentrations as low as 100 ng/ml LTA [25,27]. The human blood-based MAT has been validated for endotoxin detection but there was no formal evaluation study with respect to non-endotoxin pyrogen detection [22]. However, Hasiwa et al. [28] strongly suggested that the MAT is able to detect non-endotoxin pyrogens and whether a formal validation is necessary is beyond the scope of this discussion.

The inferior performance of the bovine assay in terms of non-endotoxin pyrogens may indicate a shortcoming. The difference in sensitivity of the two methods may partly be due to the different endpoints used. As an acute phase protein IL-1β is produced by blood cells only in response to potentially dangerous exogenous stimuli, e.g. pyrogens [29] resulting in a strong increase of its concentration. In contrast, certain endogenous levels of PGE_2_ are physiologically present in (bovine) blood. Therefore, interindividual differences in basal plasma levels demanded a quite distinct increase of eicosanoid production in order to be statistically significant. Unfortunately, we were unable to obtain a commercial kit suitable for the determination of cytokines in bovine whole blood [14]. Future investigations will need to clarify whether the bWBA can be optimized in order to increase its sensitivity, but the fact that the level of significance was just barely missed after stimulation with 1 μg/ml LTA seems promising.

Some medicinal products like vaccines may benefit from in vitro pyrogen testing in the target species. Bacterial vaccines contain bacterial components by definition and different species may display differing sensitivities towards certain bacteria [30]. Thus, testing vaccines in the target species may increase product safety. Considering the diversity of veterinary species it will, however, be very difficult to test every product in the target species, especially because many human medicinal products (with the exception of vaccines) are used off-label in veterinary medicine. With that said, pyrogen testing using quality-controlled blood from cattle housed under standardized conditions may be an option with favorable risk/benefit-ratio.

## Conclusions

With regard to the aim of reduction, refinement and replacement (3R) of animal experiments the introduction of the MAT seemed promising for the reduction of the use of RPT and LAL [31]. However, this objective does not seem to have been achieved because the MAT is not widely used yet. Here we show the potential of detecting endotoxin and non-endotoxin pyrogens using a bovine whole blood assay. Further efforts are indispensable to improve the method’s functionality, detection limits and robustness as well as to verify whether it can detect further pyrogens including lipopeptides. If the bWBA meets these requirements it should be possible to produce large standardized batches of bovine blood in reference laboratories which could then offer pyrogen testing services using the bWBA as an alternative to the RPT.

## Endnote

^1^Notably, concentrations in the figures refer to the stimulant solutions used. These were diluted tenfold in the final setup (using 225 μl whole blood and 25 μl stimulant). Results depicted in the figures are representative of repeated experiments. The detection limits from all experiments are presented in Table 1.

**Table 1:** Detection limits of the different stimulants

	**Detection limits**			
	**Experiment 1**	**Experiment 2**	**Experiment 3**	**PyroDetect System**
	**n = 6 – 9**	**n = 6**	**n = 5**	**Poolblood**
LPS *E.coli*	0.08 EU/ml	0.04 EU/ml	0.08 EU/ml	0.04 EU/ml ‡
0127:B8				
LPS *E.coli*	not tested	0.5 EU/ml	0.25 EU/ml	0.25 EU/ml
0113:H10				
Peptidoglycan	10 μg/ml	50 μg/ml	10 μg/ml	1 μg/ml ‡
*Bac. subtilis*				
Lipoteichoic acid	10 μg/ml	10 μg/ml	10 μg/ml	1 μg/ml ‡
*Staph. aureus*				

Different animals were used, permutation test, compared to unstimulated blood. Experiment 3 was the comparison with the PyroDetect System. ‡ = lowest concentration tested.

## Abbreviations

bWBA: Bovine whole blood assay; MAT: Monocyte activation test; RPT: Rabbit pyrogen test; LAL: Limulus amoebocyte lysast test; EP: European pharmacopoeia; LPS: Lipopolysaccharid; PGN: Peptidoglycan; LTA: Lipoteichoic acid; PGE_2_: Prostaglandin E_2_; E.coli: Escherichia coli; WHO: World health organization; TLR: Toll-like receptor; EU: Endotoxin unit; NOD: Nucleotide oligomerization domain; MALP-2: Macrophage-activating lipopeptide 2.

## Competing interests

The authors declare that they have no competing interests.

## Authors’ contributions

CW, SS and MK conceived and designed the study. CW performed the experiments, CW and SS analysed the data and wrote the manuscript, MK contributed valuable discussion and critically revised the article. MK had full access to the data and is the guarantor of the study. All authors read and approved the final manuscript.

## Pre-publication history

The pre-publication history for this paper can be accessed here:

http://www.biomedcentral.com/2050-6511/15/50/prepub
